# Supplementary material for: Effect of Paxlovid treatment during acute COVID-19 on Long COVID onset: An EHR-based target trial emulation from the N3C and RECOVER consortia
Source: PLoS Med. 2025 Sep 15;22(9):e1004711. doi: 10.1371/journal.pmed.1004711 (PMC12445499; doi:10.1371/journal.pmed.1004711)
Supplement: S1 Text — Fig A: Outcome co-occurrence matrix. Each cell represents the percentage of patients with the row outcome who also had the column outcome. Fig B: Cumulative incidence of PASC in Paxlovid-treated vs. non-Paxlovid-treated patients by outcome measure; between 29 and 180 days; VA-like subanalysis. Fig C: Cumulative incidence of PASC in Paxlovid-treated vs. non-Paxlovid-treated patients by predicted outcome from CP model with threshold of 0.9 or U09.9, additionally adjusted for vaccination status and among data partners meeting vaccination data quality criteria. Fig D: Causal diagram used to inform covariate selection. Treatment is shown in green; outcome is shown in orange; observed covariates are shown in gray; unobserved covariates are shown in pink. Note that this diagram only shows the relationships relevant to this study. Covariates may have other causes that are omitted for clarity. Table A: ICD-10 codes used to define Global Burden of Disease symptom clusters. (DOCX) [file pmed.1004711.s001.docx]

# SUPPLEMENTARY MATERIAL S1

# Extended Methods

*Study Period*

The study period spanned April 1, 2022, to August 14, 2023, with an index cutoff date of February 28, 2023 (180 days before the end of the study period). We chose not to study the period between December 21, 2021 (date of Paxlovid EUA) and March 31, 2022 due to the variability in case counts and prescription patterns during the first wave of the Omicron variant.[[1]](https://paperpile.com/c/x8FP8F/W3Jk6) During the first Omicron wave, COVID-19 incidence was much higher than later in the study period, and Paxlovid treatment was much rarer. This would make it difficult to meet the positivity assumption required for causal inference, because the large majority of untreated patients would come from the first Omicron wave, and the large majority of treated patients would come from later in the study period.

*Sequential Trial Design*

The sequential trial design is a well-established approach to resolve time-related biases in causal inference.[[2,3]](https://paperpile.com/c/x8FP8F/o5pU+vghq) It is most commonly used to emulate trials with rolling admission. In our case, we employ the design to avoid introducing immortal time bias due to the five-day treatment grace period. Alternatively, a single emulated trial could categorize patients into treatment groups based on whether they received a Paxlovid prescription within five days of COVID-19 index. However, this would raise several issues. For example, how should we handle patients who died within the grace period? Hospitalization at index is an exclusion criteria - what about hospitalization within the grace period? It would be difficult to account for these issues in a single-trial design without taking post-index information into account in a patient’s treatment assignment. That would violate one of the core tenets of target trial emulation.[[4]](https://paperpile.com/c/x8FP8F/MY1i)

The sequential trial design resolves these concerns. By emulating a separate trial on each day of the grace period, and reassessing exclusion criteria for each sequential trial, we base treatment assignment only on information available at each trial’s time zero. A similar approach has been used to handle the treatment grace period in another TTE studying Paxlovid for PASC prevention.[[5]](https://paperpile.com/c/x8FP8F/8ONM)

One of the practical challenges of the sequential trial design is control group inflation. Treated patients are included in a treatment group only once, but untreated patients can be included in a control group many times. For example, a patient who never receives treatment in our study would be included in the control group six times (once for each sequential trial). Treated patients can also contribute rows to the control group prior to their treatment. This dynamic is compounded when treatment is rarer than non-treatment (as in this study). Such a design would result in a control group of 1.74 million patients in our study. This is not statistically problematic, but it would lead to far more computational expense. To mitigate control group inflation, we added the final sequential exclusion criterion: patients were excluded if they appeared in the same treatment group in a previous sequential trial. This is equivalent to downsampling the control group to each patient’s first appearance, a commonly used technique.

Sequential trial designs essentially create more than one study. As in other forms of meta-analysis, the information from these studies can be analyzed in various ways. One option is to analyze each sequential trial independently, then pool the estimates. However, this approach is not compatible with the downsampling techniques described in the previous paragraph. Instead, we pool the individual records from each study and analyze them together. This approach is akin to one-stage individual patient data meta-analysis.[[6]](https://paperpile.com/c/x8FP8F/88ma) We pool records prior to estimating propensity and censoring weights. This generates a pooled pseudo-cohort in which treatment groups are exchangeable with respect to baseline covariates and in which censoring is random with respect to treatment.

*Treatment and Outcome*

We selected a treatment grace period of 5 days from COVID-19 index to adhere as closely as possible to treatment guidelines (within 5 days of symptom onset) with the available data. We identified 10 Observational Medical Outcomes Partnership [OMOP] concepts that correspond to Paxlovid in N3C and used these concepts to measure treatment.[[7]](https://paperpile.com/c/x8FP8F/AKhSh) We chose to emulate a pragmatic target trial, in which patients who were not randomized to receive the treatment of interest could still be treated to the standard of care in all other respects.

We considered two measures of the PASC outcome. To measure PASC overall, we used a computable phenotype: a machine learning model trained to predict PASC diagnoses (ICD-10 code U09.9). An earlier version of this computable phenotype was used in prior work.[[8]](https://paperpile.com/c/x8FP8F/JDQ6g) For this study, we used an updated version better suited for the later phase of the pandemic.[[8,9]](https://paperpile.com/c/x8FP8F/G34JH+JDQ6g) The model gathers data for each patient in overlapping 100-day periods that progress through time, and issues a probability of PASC for each 100-day period. The model was trained to classify whether patients have a U09.9 (“Post COVID-19 Condition”) ICD-10 diagnosis code in each period, based on the patients’ diagnoses during each period. PASC date was defined as the start date of the 100-day period which had the maximum computable phenotype prediction above a threshold of 0.9, or, if present, the date of U09.9 diagnosis, whichever was earlier. Patients over 100 years old at COVID-19 index did not receive model scores and were excluded from analysis of this outcome.

To measure PASC at a more granular level, we examined the PASC symptom clusters--cognitive, fatigue, and respiratory--proposed by the Global Burden of Disease (GBD) Study.[[10]](https://paperpile.com/c/x8FP8F/oVnQT) These clusters were the most frequently reported symptoms in a meta-analysis of Long COVID studies. Their full definitions are cognitive problems (forgetfulness or difficulty concentrating, commonly referred to as brain fog); persistent fatigue with bodily pain (myalgia) or mood swings; and ongoing respiratory problems (primarily shortness of breath and persistent cough). For the GBD symptom cluster outcomes, we conducted two distinct types of analyses. First, we examined the effect of treatment on the onset of each GBD symptom cluster independently, with PASC date defined as the first diagnosis date of any incident symptom in the cluster at least 29 days after COVID-19 index (we defined incident symptoms as symptoms that did not occur in the three years prior to COVID-19 index). Second, we examined the effect of treatment on a composite symptom-based outcome, with PASC date defined as the earliest post-acute onset date of any incident symptom in any of the three GBD symptom clusters. The list of ICD-10 codes to define each GBD symptom cluster cluster was based on the GBD study and is presented in Table A in S1 Text. Individuals were assessed for symptom cluster outcomes regardless of their computable phenotype PASC outcomes. The symptom clusters are not mutually exclusive.

A positive prediction from the computable phenotype model does not imply that a patient must have a positive outcome for one or more symptom clusters. The model considers many more diagnosis codes than those included in the symptom clusters (see the “SNOMED Roll Up” section in the supplement of Crosskey et al., 2023), and a positive prediction may be based on other diagnosis codes.[[11]](https://paperpile.com/c/x8FP8F/m3hpg) Also, the computable phenotype model features are not restricted to incident diagnoses. For example, if a patient had a dyspnea diagnosis in the three years prior to index, a post-acute dyspnea diagnosis would not count for the respiratory symptom cluster, but it would be considered by the computable phenotype model.

*Statistical Analysis*

We used a single logistic regression model to estimate each patient’s propensity of treatment based on a set of baseline covariates. We selected covariates based on a theoretical causal model, which the author team - consisting of clinicians, epidemiologists, bioinformaticians, data scientists, and patient representatives with lived experience - developed collaboratively. Our causal model is shown as a directed acyclic graph in Supplemental Figure 4. Our specific rationale for selected covariates is as follows. Many studies have shown disparity in COVID-19 treatment and outcome by race, ethnicity, and social determinants of health.[[12–15]](https://paperpile.com/c/x8FP8F/NTJGB+hisfX+epwE2+lkX8k) Sex, age, and comorbidities are known to affect care seeking and the outcome of COVID-19. Past healthcare utilization could affect the likelihood of treatment seeking and PASC documentation. Finally, the index month was included because Paxlovid treatment rates, viral variants, and infection rates changed during the study period. CCI was coded as missing when no condition records were present in N3C prior to index. CWBI was coded as missing when patient ZIP code was not reported. Binned covariates included age, CCI, CWBI, number of visits in the year prior to index, and number of hospitalizations in the year prior to index. Age and CCI were binned into clinically meaningful categories. CWBI, number of visits, and number of hospitalizations were binned into categories chosen for a balance of interpretability and equal counts.

We used this treatment model to generate stabilized IPT weights for each individual as$proportion treated / propensity score$ for the treatment group and $proportion treated / (1 - propensity score)$ for the control group[[16]](https://paperpile.com/c/x8FP8F/CmvhC). To reduce the influence of extreme weights, we trimmed IPT weights at the 99.5th percentile. We assessed covariate balance using absolute standardized differences.

We also generated inverse probability of censoring (IPC) weights to adjust for informative censoring. Loss to follow-up was more common in the control group, which could lead to bias. IPC weighting produces a pseudo-cohort in which censoring is random with respect to treatment. To generate IPC weights, we used a single logistic regression model to estimate each patient’s propensity of censorship during the study period, based on their treatment group and the same set of covariates as the treatment model. This approach treats the relative likelihood of censoring across groups as time-invariant, an assumption that we verified by examining the cumulative incidence of censoring by treatment group over time. We used this model to generate stabilized IPC weights as$proportion censored / propensity to be censored$ for censored patients and $proportion censored / (1 - propensity to be censored)$ for uncensored patients[[17]](https://paperpile.com/c/x8FP8F/0TH01). We also trimmed IPC weights at the 99.5th percentile. Finally, we generated combined inverse probability weights as the product of IPT and IPC weights.

*Subanalyses*

In the first subanalysis, we attempted to mirror the cohort used in Xie et al (2023) to make our study more comparable to prior knowledge. In this analysis, we used the same study start and end dates as Xie et al. (January 3, 2022, and December 31, 2022). To mirror VA demographics, we filtered the cohort to males >= 65 years old at COVID-19 index.

In the second subanalysis, we included COVID-19 vaccination status as a covariate, and replicated our primary analysis. We considered vaccination to be a plausible confounder of Paxlovid treatment and documented PASC, either through acute infection severity or propensity to seek care. However, vaccination status in N3C (like most EHRs) is subject to missingness. In this subanalysis, we used a subcohort of patients from sites with reliable vaccination data, which we identified, as in prior work, by comparing each site’s data to public vaccination rates for its catchment area.[[8,18]](https://paperpile.com/c/x8FP8F/JDQ6g+6zrT7) We categorized patients by their vaccination status prior to their COVID-19 index date, defined as having completed a full course of vaccination at least 14 days prior to index. Partially vaccinated patients and patients who became fully vaccinated fewer than 14 days prior to index were excluded from the analysis.

*Sensitivity Analyses*

We conducted eight sensitivity analyses.

First, we used a doubly-robust estimation method in case the treatment model was misspecified. Computationally expensive doubly-robust methods like targeted maximum likelihood estimation were not feasible with our cohort and computing environment, so we were unable to estimate cumulative incidence using a doubly-robust method. Instead, we estimated the hazard ratio (HR) of Paxlovid treatment as a secondary estimand. We used IPT- and IPC-weighted Cox proportional hazards models adjusted for the same baseline covariates as the treatment model. The same bootstrap procedure was used to estimate confidence intervals.

Second, we tested various computable phenotype prediction thresholds. In addition to the 0.9 threshold used in the primary analysis, we tested prediction thresholds at 0.75, 0.8, 0.85, and 0.95.

Third, we included Paxlovid treatment as a COVID-19 index event. This added 10,475 additional patients who were treated with Paxlovid during the study period, but did not have a U07.1 diagnosis or a positive lab test in the five days prior to treatment.

Fourth, we also tested sensitivity to COVID-19 index definition by including only positive lab tests as index events. This removed 254,927 patients who had U07.1 diagnoses without accompanying lab results.

Fifth, we tested sensitivity to outcome definition in three ways: by requiring outcomes to occur 90 days after COVID-19 index (rather than 29 days), by observing patients for up to 365 days (rather than 180 days), and by the combination of both (observing PASC from days 90 to 365).

Sixth, we tested sensitivity to the handling of death. In the primary analysis, we treated death as a competing risk. Here, we treated death as a censoring event instead. By doing so, we estimated the direct effect of Paxlovid treatment on PASC incidence, not including any effect that is mediated by death.[[19]](https://paperpile.com/c/x8FP8F/pfJlo) Paxlovid is known to reduce mortality, and patients who would have died without Paxlovid treatment are at high risk for PASC. Therefore, Paxlovid could essentially convert some deaths into PASC, which would produce an anti-protective component of its effect. Censoring at death removes this effect from the overall effect estimate.

Seventh, we tested sensitivity to our assumption that the last visit in each patient’s EHR constitutes loss to follow-up. We did not censor patients at the date of their last documented visit in the EHR. This assumes that patients did not get care in the time after their last visit, rather than assuming that they were lost to follow-up and could have gotten care elsewhere.

Eighth, we tested sensitivity to the treatment and control arm definitions in our target trial. In the primary analysis, the target trial is pragmatic, and does not exclude patients who received other treatments. In this sensitivity analysis, we excluded patients who received two other COVID-19 treatments - Molnupiravir and Ritonavir - during the grace period from both study arms.

#

# Fig A in S1 Text: Causal diagram used to inform covariate selection. Treatment is shown in green; outcome is shown in orange; observed covariates are shown in gray; unobserved covariates are shown in pink. Note that this diagram only shows the relationships relevant to this study. Covariates may have other causes that are omitted for clarity.


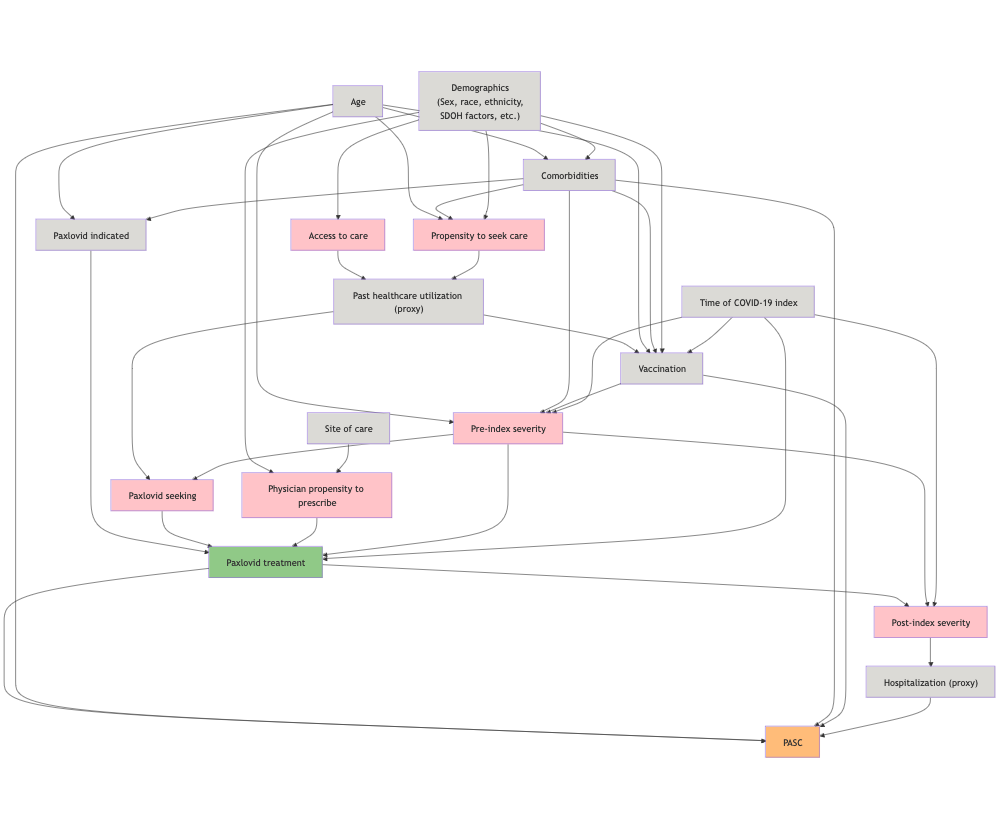


# Fig B in S1 Text: Outcome co-occurrence matrix. Each cell represents the percentage of patients with the row outcome who also had the column outcome.

#
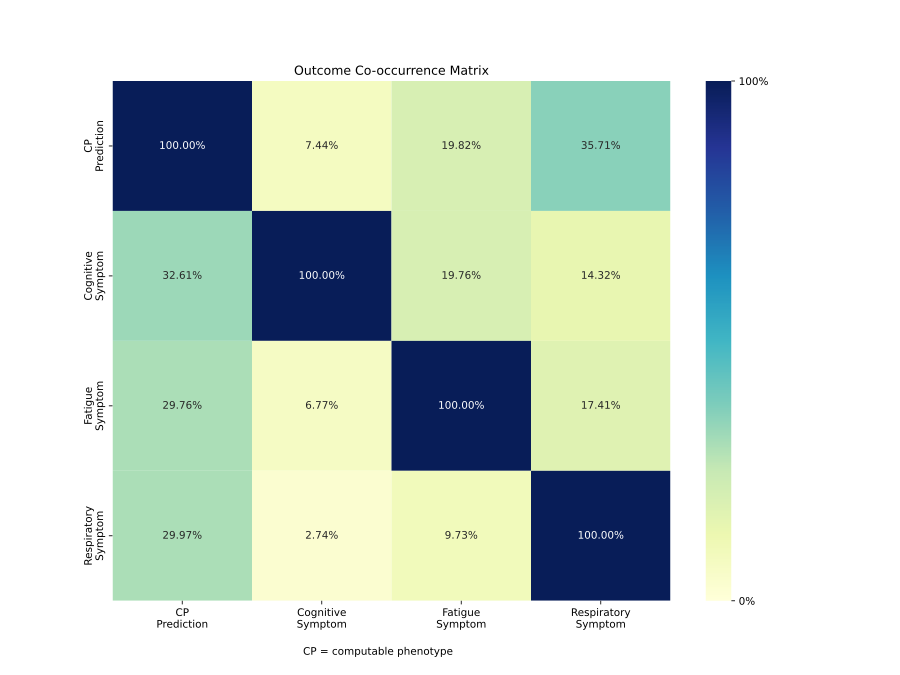


# Fig C in S1 Text: Cumulative incidence of PASC in Paxlovid treated vs. Non-Paxlovid-Treated patients by outcome measure; between 29-180 days; VA-like subanalysis

**
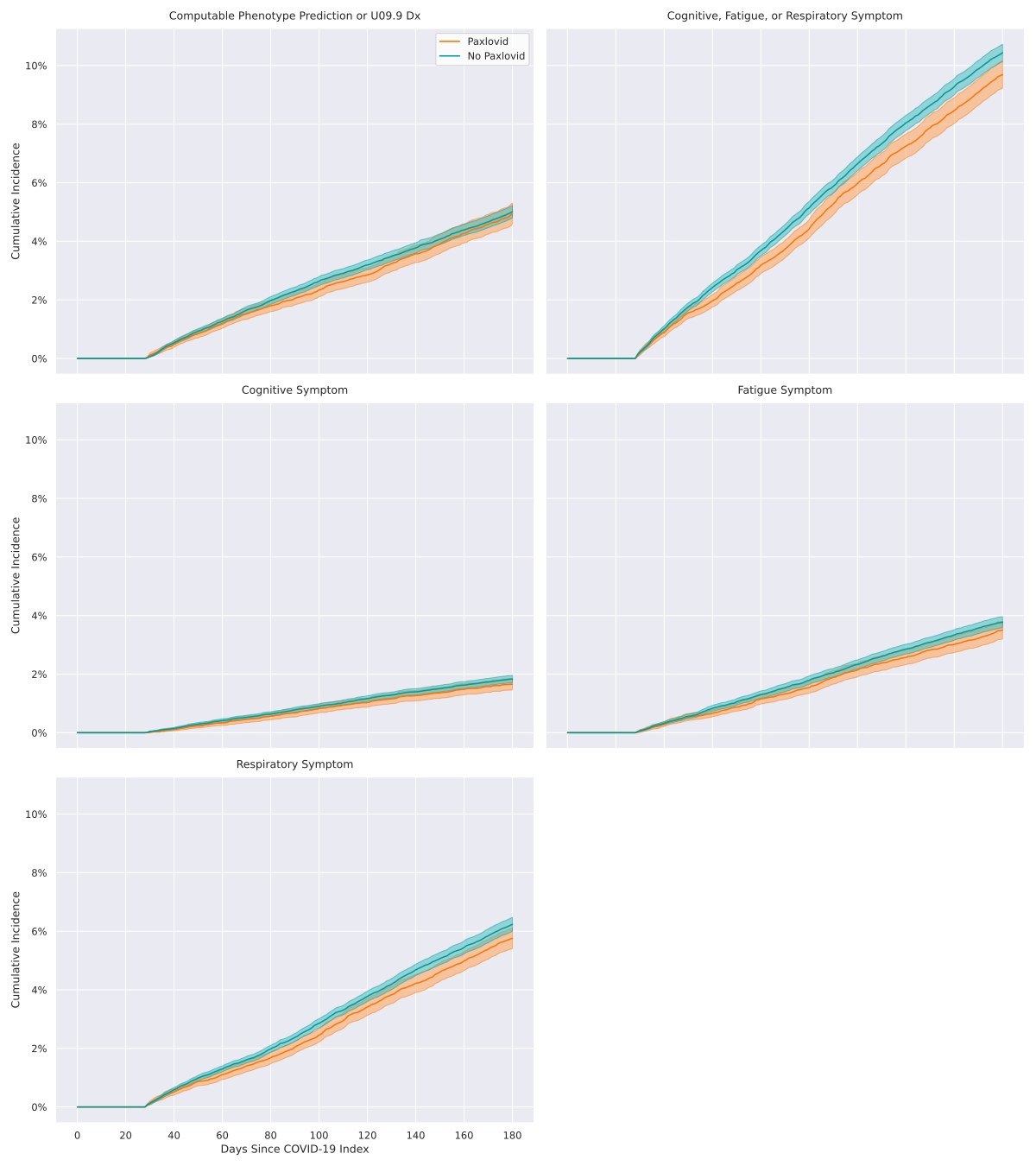
**

**Fig D in S1 Text:** **Cumulative incidence of PASC in Paxlovid treated vs. Non-Paxlovid-Treated patients by predicted outcome from CP model with threshold of 0.9 or U09.9, additionally adjusted for vaccination status and among data partners meeting vaccination data quality criteria**


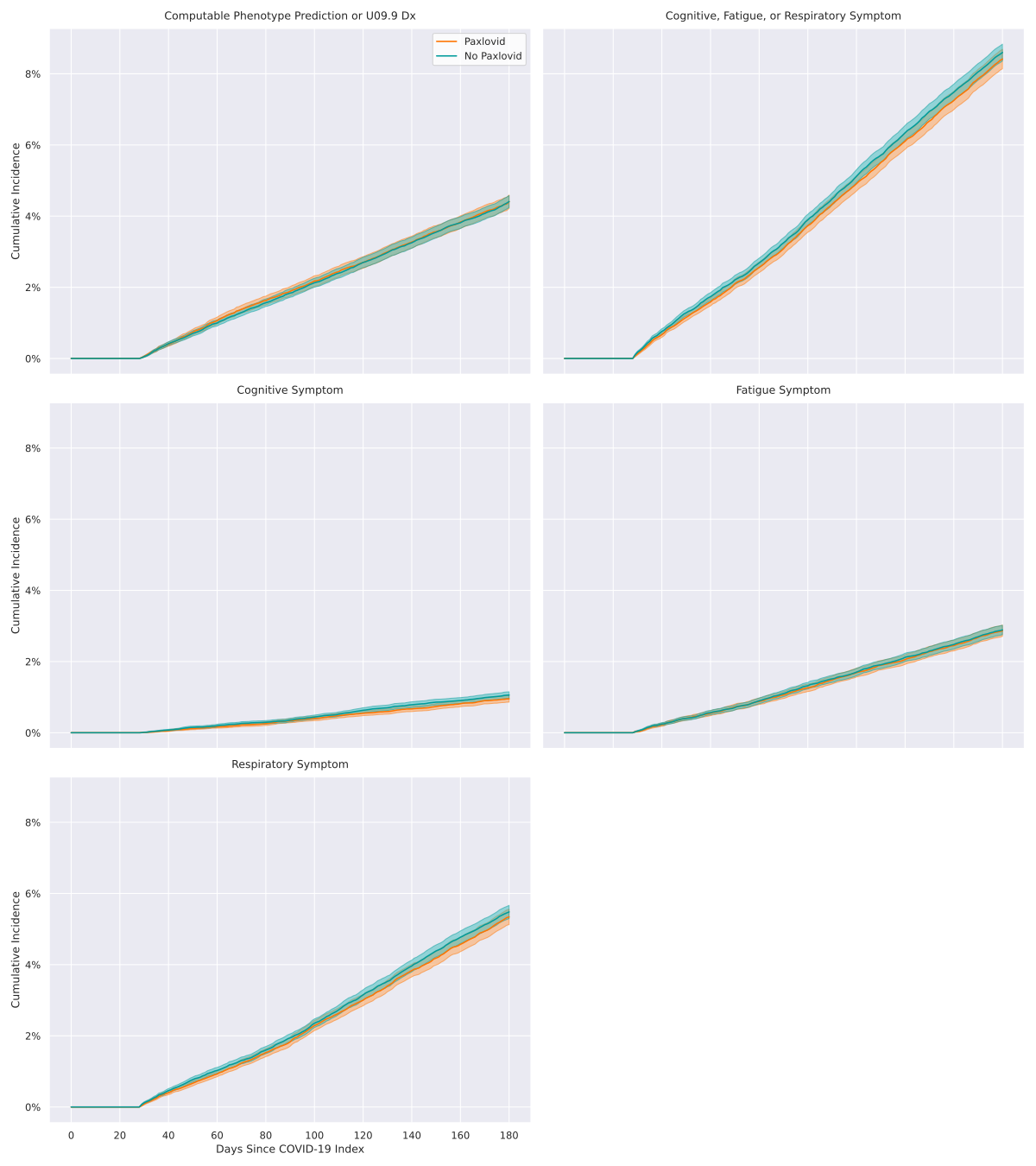


**Table A in S1 Text: ICD-10 codes used to define Global Burden of Disease symptom clusters**[**[10]**](https://paperpile.com/c/x8FP8F/oVnQT)

| **ICD-10 Code** | **ICD-10 Code Description** | **Symptom Cluster** |
| --- | --- | --- |
| R404 | Transient alteration of awareness | Cognitive |
| R410 | Disorientation unspecified | Cognitive |
| R411 | Anterograde amnesia | Cognitive |
| R412 | Retrograde amnesia | Cognitive |
| R413 | Other amnesia | Cognitive |
| R4182 | Altered mental status unspecified | Cognitive |
| R41840 | Attention and concentration deficit | Cognitive |
| R41841 | Cognitive communication deficit | Cognitive |
| R4189 | Other symptoms and signs involving cognitive functions and awareness | Cognitive |
| R419 | Unspecified symptoms and signs involving cognitive functions and awareness | Cognitive |
| R531 | Weakness | Fatigue |
| R5381 | Other malaise | Fatigue |
| R5382 | Chronic fatigue unspecified | Fatigue |
| R5383 | Other fatigue | Fatigue |
| J9610 | Chronic respiratory failure unspecified whether with hypoxia or hypercapnia | Respiratory |
| J9611 | Chronic respiratory failure with hypoxia | Respiratory |
| J9612 | Chronic respiratory failure with hypercapnia | Respiratory |
| J9620 | Acute and chronic respiratory failure unspecified whether with hypoxia or hypercapnia | Respiratory |
| J9621 | Acute and chronic respiratory failure with hypoxia | Respiratory |
| J9622 | Acute and chronic respiratory failure with hypercapnia | Respiratory |
| J9690 | Respiratory failure unspecified unspecified whether with hypoxia or hypercapnia | Respiratory |
| J9691 | Respiratory failure unspecified with hypoxia | Respiratory |
| J9692 | Respiratory failure unspecified with hypercapnia | Respiratory |
| J988 | Other specified respiratory disorders | Respiratory |
| J989 | Respiratory disorder unspecified | Respiratory |
| J99 | Respiratory disorders in diseases classified elsewhere | Respiratory |
| R05 | Cough | Respiratory |
| R0600 | Dyspnea unspecified | Respiratory |
| R0602 | Shortness of breath | Respiratory |
| R0603 | Acute respiratory distress | Respiratory |
| R0609 | Other forms of dyspnea | Respiratory |
| R071 | Chest pain on breathing | Respiratory |

#

# References

1. [Chatterjee S, Bhattacharya M, Nag S, Dhama K, Chakraborty C. A Detailed Overview of SARS-CoV-2 Omicron: Its Sub-Variants, Mutations and Pathophysiology, Clinical Characteristics, Immunological Landscape, Immune Escape, and Therapies. Viruses. 2023;15. doi:](http://paperpile.com/b/x8FP8F/W3Jk6)[10.3390/v15010167](http://dx.doi.org/10.3390/v15010167)

2. [Dickerman BA, García-Albéniz X, Logan RW, Denaxas S, Hernán MA. Avoidable flaws in observational analyses: an application to statins and cancer. Nature medicine. 2019;25. doi:](http://paperpile.com/b/x8FP8F/o5pU)[10.1038/s41591-019-0597-x](http://dx.doi.org/10.1038/s41591-019-0597-x)

3. [García-Albéniz X, Hsu J, Hernán MA. The value of explicitly emulating a target trial when using real world evidence: an application to colorectal cancer screening. Eur J Epidemiol. 2017;32: 495–500. doi:](http://paperpile.com/b/x8FP8F/vghq)[10.1007/s10654-017-0287-2](http://dx.doi.org/10.1007/s10654-017-0287-2)

4. [Hernán MA, Sauer BC, Hernández-Díaz S, Platt R, Shrier I. Specifying a target trial prevents immortal time bias and other self-inflicted injuries in observational analyses. Journal of clinical epidemiology. 2016;79. doi:](http://paperpile.com/b/x8FP8F/MY1i)[10.1016/j.jclinepi.2016.04.014](http://dx.doi.org/10.1016/j.jclinepi.2016.04.014)

5. [Ioannou GN, Berry K, Rajeevan N, Li Y, Mutalik P, Yan L, et al. Effectiveness of nirmatrelvir-ritonavir against the development of post-COVID-19 conditions among U.s. veterans : A target trial emulation: A target trial emulation. Ann Intern Med. 2023;176: 1486–1497. doi:](http://paperpile.com/b/x8FP8F/8ONM)[10.7326/M23-1394](http://dx.doi.org/10.7326/M23-1394)

6. [Veroniki AA, Seitidis G, Tsivgoulis G, Katsanos AH, Mavridis D. An introduction to individual participant data meta-analysis. Neurology. 2023;100: 1102–1110. doi:](http://paperpile.com/b/x8FP8F/88ma)[10.1212/WNL.0000000000207078](http://dx.doi.org/10.1212/WNL.0000000000207078)

7. [Hripcsak G, Duke JD, Shah NH, Reich CG, Huser V, Schuemie MJ, et al. Observational Health Data Sciences and Informatics (OHDSI): Opportunities for Observational Researchers. Stud Health Technol Inform. 2015;216: 574–578. doi:](http://paperpile.com/b/x8FP8F/AKhSh)[10.1038/psp.2013.52](http://dx.doi.org/10.1038/psp.2013.52)

8. [Brannock MD, Chew RF, Preiss AJ, Hadley EC, Redfield S, McMurry JA, et al. Long COVID risk and pre-COVID vaccination in an EHR-based cohort study from the RECOVER program. Nat Commun. 2023;14: 2914. doi:](http://paperpile.com/b/x8FP8F/JDQ6g)[10.1038/s41467-023-38388-7](http://dx.doi.org/10.1038/s41467-023-38388-7)

9. [Pfaff ER, Girvin AT, Bennett TD, Bhatia A, Brooks IM, Deer RR, et al. Identifying who has long COVID in the USA: a machine learning approach using N3C data. Lancet Digit Health. 2022;4: e532–e541. doi:](http://paperpile.com/b/x8FP8F/G34JH)[10.1016/S2589-7500(22)00048-6](http://dx.doi.org/10.1016/S2589-7500(22)00048-6)

10. [Global Burden of Disease Long COVID Collaborators, Wulf Hanson S, Abbafati C, Aerts JG, Al-Aly Z, Ashbaugh C, et al. Estimated Global Proportions of Individuals With Persistent Fatigue, Cognitive, and Respiratory Symptom Clusters Following Symptomatic COVID-19 in 2020 and 2021. JAMA. 2022;328: 1604–1615. doi:](http://paperpile.com/b/x8FP8F/oVnQT)[10.1001/jama.2022.18931](http://dx.doi.org/10.1001/jama.2022.18931)

11. [Crosskey M, McIntee T, Preiss S, Brannock D, Yoo YJ, Hadley E, et al. Reengineering a machine learning phenotype to adapt to the changing COVID-19 landscape: A study from the N3C and RECOVER consortia. bioRxiv. 2023. doi:](http://paperpile.com/b/x8FP8F/m3hpg)[10.1101/2023.12.08.23299718](http://dx.doi.org/10.1101/2023.12.08.23299718)

12. [Klein EJ, Hardesty A, Vieira K, Farmakiotis D. Use of anti-spike monoclonal antibodies in kidney transplant recipients with COVID-19: Efficacy, ethnic and racial disparities. Am J Transplant. 2022;22: 640–645. doi:](http://paperpile.com/b/x8FP8F/NTJGB)[10.1111/ajt.16843](http://dx.doi.org/10.1111/ajt.16843)

13. [Wiltz JL, Feehan AK, Molinari NM, Ladva CN, Truman BI, Hall J, et al. Racial and Ethnic Disparities in Receipt of Medications for Treatment of COVID-19 - United States, March 2020-August 2021. MMWR Morb Mortal Wkly Rep. 2022;71: 96–102. doi:](http://paperpile.com/b/x8FP8F/hisfX)[10.15585/mmwr.mm7103e1](http://dx.doi.org/10.15585/mmwr.mm7103e1)

14. [Wu E-L, Kumar RN, Moore WJ, Hall GT, Vysniauskaite I, Kim K-YA, et al. Disparities in COVID-19 Monoclonal Antibody Delivery: a Retrospective Cohort Study. J Gen Intern Med. 2022;37: 2505–2513. doi:](http://paperpile.com/b/x8FP8F/epwE2)[10.1007/s11606-022-07603-4](http://dx.doi.org/10.1007/s11606-022-07603-4)

15. [Boehmer TK, Koumans EH, Skillen EL, Kappelman MD, Carton TW, Patel A, et al. Racial and Ethnic Disparities in Outpatient Treatment of COVID-19 - United States, January-July 2022. MMWR Morb Mortal Wkly Rep. 2022;71: 1359–1365. doi:](http://paperpile.com/b/x8FP8F/lkX8k)[10.15585/mmwr.mm7143a2](http://dx.doi.org/10.15585/mmwr.mm7143a2)

16. [Chesnaye NC, Stel VS, Tripepi G, Dekker FW, Fu EL, Zoccali C, et al. An introduction to inverse probability of treatment weighting in observational research. Clin Kidney J. 2021;15: 14–20. doi:](http://paperpile.com/b/x8FP8F/CmvhC)[10.1093/ckj/sfab158](http://dx.doi.org/10.1093/ckj/sfab158)

17. [Hernan MA, Robins JM. Causal inference: What if. Boca Raton, FL: CRC Press; 2024. Available:](http://paperpile.com/b/x8FP8F/0TH01) <https://books.google.com/books/about/Causal_Inference.html?id=_KnHIAAACAAJ>

18. [Bhatia A, Preiss AJ, Xiao X, Brannock MD, Alexander GC, Chew RF, et al. Effect of Nirmatrelvir/Ritonavir (Paxlovid) on Hospitalization among Adults with COVID-19: an EHR-based Target Trial Emulation from N3C. medRxiv. 2023. doi:](http://paperpile.com/b/x8FP8F/6zrT7)[10.1101/2023.05.03.23289084](http://dx.doi.org/10.1101/2023.05.03.23289084)

19. [Young JG, Stensrud MJ, Tchetgen Tchetgen EJ, Hernán MA. A causal framework for classical statistical estimands in failure-time settings with competing events. Stat Med. 2020;39: 1199–1236. doi:](http://paperpile.com/b/x8FP8F/pfJlo)[10.1002/sim.8471](http://dx.doi.org/10.1002/sim.8471)
