# Supplementary material for: Effect of Paxlovid treatment during acute COVID-19 on Long COVID onset: An EHR-based target trial emulation from the N3C and RECOVER consortia
Source: PLoS Med. 2025 Sep 15;22(9):e1004711. doi: 10.1371/journal.pmed.1004711 (PMC12445499; doi:10.1371/journal.pmed.1004711)
Supplement: Attachment — Submitted filename: 02_Paxlovid PASC PLOSMed Response to Reviewers (Sept 2024).pdf [file pmed.1004711.s005.pdf]

Academic Editor:

I think a major revision is appropriate.

1. It does seem that the authors used the TTE and cloning-censoring-weighting approach as they did with the last manuscript. However, the aspect of cloning and censoring is not well described.

    We did not use the clone-censor-weight technique in this study, because the treatment grace period (days 0-5) and the outcome observation period (days 29-180) do not overlap. In other words, all clones would be censored by the time the outcome is possible. Because immortal time bias is not a concern in this study design, we used IPTW instead to establish exchangeability. In this revision, we also added IPCW to account for the possibility of informative censoring due to differences in rates of loss to follow-up and death across arms.

2. I think there is potential for bias to exclude individuals who were hospitalized prior to receipt of Paxlovid from the treatment arm. In a hypothetical RCT where treatment is assigned on the date of diagnosis, individuals who are hospitalized prior to treatment with paxlovid would still be considered in the treatment arm (if the outcome is PASC). In this case, being hospitalized becomes a competing risk a precludes some individuals from being in treatment arm but not control.

    We believe that the fundamental issue here was a lack of clarity in the treatment and control regimen definitions. We added more detail and specificity to the definitions in Table 2. We specified that our treatment of interest is Paxlovid given in the outpatient setting, and neither (a) Paxlovid treatment in the inpatient setting, nor (b) Paxlovid treatment after 5 days, preclude patients from being in the control arm. This makes our target trial pragmatic, in the sense that patients who were not randomized to receive the treatment of interest could still be treated to the standard of care in all other respects, including the possible prescription of Paxlovid in ways that do not meet the treatment definition.

    To handle these more specific treatment and control definitions, we did the following.

    1. As in the initial submission, patients who received Paxlovid within 5 days, but during a hospitalization, were placed in the control group. In target trial terms, we treat these patients as if they were not randomized to receive treatment, and were pragmatically given Paxlovid as inpatients while in the control arm.

2. We no longer censor this group of patients when they receive Paxlovid. This is because the control regimen definition does not preclude other forms of Paxlovid prescription that do not meet the treatment definition.
3. We also no longer censor patients in the control arm if they were prescribed Paxlovid after day 5, for the same reason. (This does not directly affect your point about hospitalization as a competing risk, but it is worth mentioning in this context, because we also based this action on the updated regimen definitions.)

3. Death is certainly a competing risk but authors still chose to censor. I was not clear of the explanation on how competing risk is considered a mediator. In this circumstance, it could be particularly relevant to treat appropriately as competing risk. Theoretically, Paxlovid could reduce mortality, but those in whom death was prevented could be at much higher risk of PASC. In that sense, Paxlovid could essentially be converting deaths into alive, but PASC. Understanding that full picture with the appropriate use of competing risk seems relevant to understand incidence of both. Fine gray models could be used for measuring HR.

    We clarified the explanation on why we think treating death as a censoring event leads to a more practical interpretation of the results. The potential "converting deaths into PASC" component of Paxlovid's effect is exactly what we intended to avoid in our estimate of Paxlovid's direct effect, and why we chose to censor on death. However, we agree that understanding a full picture of this mechanism would help the reader. We also added a sensitivity analysis in which we treat death as a competing risk. Because death is so rare in this cohort, PASC incidence estimates were effectively unchanged (0.1 percentage points lower in each arm).

4. I think more discussion on the importance and relevance of using the phenotypic model for the outcome is important. Initially, I wanted to see a sensitivity analysis where they only used documented ICD 10 codes as the outcome. But then I saw that in the results, which interesting showed more PASC with treatment. In hindsight, this might make sense as people with more healthcare access are likely to get Paxlovid and evaluated for PASC. I think a discuss of the pros adn cons is important. Initially, I was wondering whether it added additional complexity and opaqueness without a clear benefit. This issue should be addressed clearly (it may be in the cited papers but some discussion needs to be in methods as well).

    We added further discussion of this topic to the methods section. We mention specific weaknesses of alternative outcome measures and how the computable phenotype avoids those problems.

5. In comparison with Xie et al, would also want some comments on similarities/differences in the approaches taken. It states in discussion that methodology is similar but I couldn't see much beyond that. If authors analyzed the VA dataset using

the same method they use here, would they anticipate getting same results as Xie et al or their own? Based on discussion, they seem to be assuming that they would get same results as Xie et al as they attribute differences mostly to the population.

> We revised our comparison of our VA-like subanalysis to Xie et al. We argue that methodological differences are unlikely to cause the different findings, particularly because our unadjusted effects are smaller for two outcomes that are very similar across the studies. To put it in your terms, we do think that applying our methods to the VA dataset would likely produce results similar to theirs.

6. Stratified estimates could also be of potential interest. Particular based on age, vaccination status(i.e., effect in vaccinated and not vaccinated population, not adjusting for vaccination).

> Although we agree that stratified estimates would be interesting, due to the complexity of the other revisions suggested by the reviewers, we were unable to add this analysis without delaying our turnaround time excessively.

Reviewer #1:

Thank you for the opportunity to review this manuscript. Overall, the authors made a very good attempt to answer the question at hand, but there some pertinent points that need addressing.

1. The manuscript is too long. As an insight, the introduction is nearly 2 pages and discussion is nearly 4 pages. There is also a lot of repetition. To make it easier to read, considerable effort should be made to write in a concise manner. A lot of the detail could be moved to an appendix.

    > We edited the introduction and discussion for brevity. The introduction is now 1.5 pages and the discussion is just over 3 pages. We also added a methods supplement, which enabled us to reduce the methods section from 7 to 2.5 pages. Although the paper is still relatively long, we believe the remaining content is necessary to present and discuss the many analyses that constitute this study. There was substantial repetition in the way the initial submission was structured, and we appreciate this suggestion, which greatly improved the flow of the paper.

2. The treatment strategies of interest are unclear. In Table 3, the treatment strategy for only 1 arm is outlined: the paxlovid arm. Even within this description, there is no information on how long treatment should be taken for, or if patients are allowed to stop for any reason. There should then additionally be detailed information on the treatment strategy for the "no treatment" arm. Should patients continue not to take treatment for the duration of follow up? Reading the text, it seems this is the case as individuals that are not prescribed at baseline are censored if the start treatment at a later date. However, all this information should be made clear in the definition of the treatment strategies.

    > Thank you for pointing out this issue. We added a control arm definition and added more detail to the treatment arm definition in Table 2 (previously Table 3).
    >
    > In the treatment arm, we clarified that we do not require any specific duration of treatment or monitoring of adherence. The provider's intention to treat, in the form of prescribing outpatient Paxlovid within the grace period, is sufficient for inclusion in the treatment arm.
    >
    > We also changed our censoring approach to align better with an ITT effect, and defined the control arm with this in mind. Please see our response to the academic editor's comment 2 and your comment 3 for details.

3. Follow on from above, the causal contrast row in Table 3 states that the investigators aim to estimate the intention-to-treat effect. This is the effect of assignment to one of the treatment arms at baseline, regardless of what happens later. However, in the "no treatment" group, they censor when someone starts treatment. This, therefore, is not akin to an ITT effect. If the investigators do censor at initiation in the no treatment arm, then they should also censor at non-adherence to the treatment strategy in the treatment arm.

This would then be more similar to a per protocol effect and would also requite adjusting for baseline and time-updated predictors of non-adherence. But, this also requires stricter definition of what the treatment strategies are, as specified above.

> We agree that our previous approach to censoring did not align with our goal to estimate an ITT effect. We no longer censor patients in the control group who are prescribed Paxlovid in ways that do not meet the treatment definition (as inpatients or >5 days after index). We specify this in the control arm definition that we added to Table 2. This makes our target trial pragmatic, in the sense that patients who were not randomized to receive the treatment of interest could still be treated to the standard of care in all other respects, including the possible prescription of Paxlovid in ways that do not meet our treatment definition.

4. There is a misalignment between assignment and when outcomes start to be collected. Treatment with paxlovid is assessed within 5 days after COVID diagnosis, but individuals are also censored if they have an event within the first 28 days after COVID. So, outcomes only start to be counted after 28 days. Why is this? It leave the study prone to immortal time.

> We begin assessing PASC at day 29 to avoid conflating acute symptoms with PASC. Most PASC studies do this; some wait up to 3 months to begin considering symptoms to be post-acute. The immortal time period from days 0 to 28 is the same regardless of treatment assignment, so it does not lead to immortal time bias.
>
> However, censoring on PASC predictions or diagnoses during the period before outcome assessment was a flaw in the initial study design. In the revision, we now ignore PASC predictions or diagnoses during this period. I.e., patients with a PASC prediction or diagnosis between days 0 and 28 remain in the study and at risk to have a PASC prediction or diagnosis from day 29 on.

5. Follow up can end at the time of the last recorded visit? Please can the investigators expand on what this means?

> Some patients have their last EHR record during the study period. This may be because they did not get additional care or because they got care at another health system. The proportions are unknown. Therefore, there is no “correct” way to handle these patients. They could either be censored (in which case we assume that they were lost to follow-up) or treated as remaining at risk and not having the outcome (in which case we assume that they did not get additional care). We added a sensitivity analysis in which we do not censor at the time of the last recorded visit. Palxovid’s effect size was smaller than in the main analysis, which suggests that the study is somewhat sensitive to this assumption. But our bottom-line conclusion would not be changed.

6. As paxlovid is known to reduce the risk of hospitalization, hospitalization could be a "semi-competing event". This is because people that are hospitalized may receive other care

and treatments that in turn reduce their risk of PASC. If those with paxlovid are less likely to be hospitalized, then the effect estimated in this study is not interpretable. Please can the investigators clarify how hospitalization is dealt with, and how they have handled the possibility this this is a competing event.

Several studies have found that more severe acute infection or hospitalization is associated with a higher risk of PASC (see references 14-17 in the manuscript). Given that Paxlovid reduces the risk of hospitalization due to COVID-19, this makes hospitalization a mediator in the causal chain between Paxlovid and PASC. Therefore, conditioning on hospitalization would remove any component of Paxlovid's effect on PASC that is mediated by hospitalization/acute disease severity. Treating hospitalization as a censoring event or competing risk would also be inappropriate, because hospitalization does not make PASC unobservable or make the patient no longer at risk for PASC. For these reasons, we chose to treat hospitalization as a mediator and a proxy for post-index COVID-19 severity. We show this in our causal model (Supplemental Figure 4).

7. How was missing data handled?

Missingness is shown in Table 1. Most of the variables used in the treatment and censoring models had low levels of missingness, with the exception of Community Wellbeing Index (CWBI) and Charlson Comorbidity Index (CCI). CWBI was coded as missing when the patient's residential ZIP code was unreported, and CCI was missing if no pre-index condition exposures were recorded (if the patient had no prior visits recorded). These variables were binned for the treatment and censoring models, so we handled missingness by adding a missing category. Vaccination data missingness varied by site, unlike the other variables, which generally had more consistent missingness across sites. We handled this by adding the vaccination sub-analysis, in which we limited the cohort to sites with high-quality vaccination data.

8. Please run the analyses with at least 500 bootstrap samples.

We reran the primary analysis with 500 bootstrap samples, and confidence intervals for PASC cumulative incidence were identical at two decimal places. To save time, we chose not to rerun other analyses with more bootstrap samples.

9. The discussion states that using the target trial framework allowed to account for confounding. This is not what the target trial framework does. It is a framework to formalize study design, but the only way to account for confounding it to adjust analyses for baseline variables.

We updated this sentence to "Our use of the TTE framework with IPTW and IPCW allowed us to account for confounding and informative censoring and to estimate the causal effect of Paxlovid treatment using observational data".

Reviewer #2:

Thank you for this opportunity to review this article reporting a study emulating a target trial on Paxlovid in association with a hypothesized lower risk of PASC, i.e., long Covid.

I find the writing to be clear and organized, with results presented comprehensively. There are the following points I would like to raise for the authors' consideration:

1. The index date was set at Covid diagnosis or positive SARS-CoV-2 test. What if the person gets Covid and was about to be prescribed Paxlovid within 5 days but didn't because they were admitted to the ICU or unconscious? What if the person dies 2 days of Covid diagnosis and did not make it to receive Paxlovid prescription? Would these individuals cause bias to the results and how did the authors address this? Why did the authors' not use cloning-censoring-weighting, for which Miguel Hernan is highly supportive, to address this challenge? Also see this example for your reference: https://doi.org/10.7326/M22-3057

   We have used the clone-censor-weight technique in other studies, including a study of Paxlovid's effect on hospitalization that is in press at PLOS Medicine. In this study, we chose not to, because the treatment grace period and the period of outcome observation do not overlap, so there is no immortal time bias. In other words, all clones would be censored by the time the outcome is possible. Instead, we used IPTW to establish exchangeability at time zero. In this revision, we also added IPCW to account for the possibility of informative censoring due to differences in rates of loss to follow-up and death across arms.

   There are likely some patients in the control group who would have been prescribed Paxlovid as outpatients (i.e., been in the treatment group), but did not because they were hospitalized. This is a potential source of bias inherent to retrospective data - we can think of it as error in the measurement of the provider's intention to treat. Cloning would not remove the bias, because clones of these patients in the treatment group would be censored at the time of hospitalization, which is prior to the period of outcome observation. Regardless, any bias caused by this dynamic would make Paxlovid's effect appear more protective than reality, because hospitalized patients (who have high risk of PASC) would be placed in the control group when they should be in the treatment group. Because we did not find a significant effect of Paxlovid on PASC, any bias in that direction would not affect our conclusion.

   We treat death as a censoring event in our primary analysis (see our response to the academic editor's comment 3). IPCW addresses any bias from informative censoring. We also conducted a sensitivity analysis with death as a competing risk and results were similar.

2. Maybe I missed it but I did not see methods describing the approach to address other antivirals, immunotherapy or other pharmacotherapies. Hence, the non-use group is a diverse group of people, possibly using different therapies. For example, remdesivir was quite commonly used for Covid for a while. What about molunupiravir?

    In the primary analysis, we did not control for or exclude patients who receive other therapies. In this respect, we emulate a pragmatic target trial, where treatment assignment does not affect any other aspect of the standard of care. To clarify this, we added more detail and specificity to the treatment and control regimen definitions shown in Table 2.

    In the revision, we also added a sensitivity analysis in which we excluded patients who received Remdesivir or Molnupiravir during the five-day treatment assignment grace period. Unsurprisingly, this was more common in the control group (4%) than in the treatment group (1%). Palxovid's effect size was smaller than in the main analysis, which suggests that the study is somewhat sensitive to this aspect of the target trial protocol. But our bottom-line conclusion would not be changed.

3. Hazard ratio is roughly an average of the ratios of hazard rates between the exposed and unexposed groups across the observation period, but only considering the surviving cohort at each time point. In a clinical trial, which this study aims to emulate, hazard ratios are not commonly used because randomization is only useful if the comparison is between the groups in full.

    We agree that hazard ratios are not appropriate causal contrasts. For this reason, we used cumulative incidence estimates, and the risk ratios between them, as our primary causal contrast. We only use hazard ratios in the sensitivity analysis where we implement a doubly robust estimator. As reviewer 4 points out, this is not ideal, because hazard rates and risk ratios are not comparable. Unfortunately, we were unable to implement compute-intensive doubly robust methods that are capable of estimating cumulative incidence in the N3C Enclave, so we were forced to accept this limitation.

Reviewer #3:

I think this is a high-quality analysis with detailed and careful reporting that will be of interest to the journal readers who may be interested in the methodology or the generated evidence. I have a few major comments as summarized below.

Major comments

1. It would be great if not essential to replicate the results of an RCT first before moving on to estimate effects that are not yet evaluated by trials. The authors have mentioned a few of those possible trials in the Introduction. I suppose it is also possible to replicate the effect of Paxlovid on mortality. Another option would be to choose a negative control outcome.

    We have another study in press at PLOS Medicine in which we estimate Paxlovid's effect on hospitalization and death (https://pubmed.ncbi.nlm.nih.gov/37205340/). Our findings were consistent with other real-world studies. (Paxlovid's real-world effect on these outcomes seems to be substantially smaller than its effect in clinical trials.)

2. If #1 is doable, I would replace the VA analysis with this new evidence as replicating another observational analyses with a new one is not as informative. As the authors have correctly mentioned, there is always the possibility of effect measure modification in addition to confounding which makes the results much less interesting.

    Our intent with the VA-like subanalysis was not to demonstrate our ability to replicate other observational studies, but rather to explore why our primary findings were so different from those of Xie et al. We clarified the purpose of the subanalysis in the method section where it is introduced, and we revised our discussion to specify why we think the findings suggest that cohort differences explain most of the difference in findings across these studies.

3. The estimated effects on cognitive function and fatigue are rather small. The authors have labeled similar effects in other analyses as practically insignificant. I suggest using the same qualification for these effects in the Abstract, Results and Discussion, especially considering that as the authors have mentioned, these are rather uncommon outcomes.

    We now avoid the term "practical significance" and instead focus on quantitative measures like absolute risk difference and number needed to treat.

4. I would suggest revising the causal graph by removing the box from the nodes that are not directly adjusted for in the analyses. That will allow the readers to identify potential bias pathways.

We changed the shape of unobserved nodes and explained the meaning of different shapes in the figure title.

Minor comments

1. There are too many tables and figures. I suggest moving Figures 4, and 7-10 as well as Tables 1 and 5 to the online Appendix.

   We moved several figures to the supplement and removed several others entirely.

2. Please clarify if the protocol was registered before conducting the analyses.

   It was not. We added mention of this to the Ethics Approval section.

3. Page 12, much of the second paragraph can be moved to the Methods.

   Thank you for catching this. We have removed the text from this paragraph that was repetitive of the methods section.

Reviewer #4:

I have a number of comments that I hope the Editor and Authors will be find helpful:

Major Comments

1. Abstract: control treatment is not defined or mentioned

   We added specific definitions of the treatment and control groups to the abstract.

2. Outcome

a. Symptom clusters were defined based on 'novel onset of PASC symptoms'. Please specify if only individuals with PASC based on the algorithm were assessed for symptoms-specific outcomes. Please specify what 'novel' means and if the clusters are mutually exclusive or if the same individuals could contribute to more than one symptom cluster.

   Individuals were assessed for symptom cluster outcomes regardless of their computable phenotype PASC outcomes. The symptom clusters are not mutually exclusive. We have clarified this in the methods section.

b. I find it difficult to understand why individuals hospitalized within 5 days of COVID before treatment were censored while individuals who were hospitalized within 5 days of COVID and had no treatment were not censored. This can introduce bias.

   We adjusted our approach to censoring to remove this source of bias. Please see our response to the academic editor's comment 2 for details.

c. Given that Paxlovid is indicated for individuals with moderate or severe COVID-19 symptoms, it is unclear why individuals who were hospitalized on the day of diagnosis were excluded.

   Paxlovid is indicated for mild-to-moderate COVID-19 in adults who are at high risk for progression to severe COVID-19, including hospitalization or death. Paxlovid is used off-label among inpatients, but this constitutes a distinct treatment modality, and our goal was to assess the effect of on-label Paxlovid use on PASC.

d. A treatment window/grace period of 5 days was used based on guidelines and this seems a reasonable choice. However, in real-world clinical practice, Paxlovid treatment might have been delayed beyond the 5-day time window in some individuals who then be classified as controls. Please present the distribution of time (median [IQR]) between COVID-19 index date and treatment initiation to show that the majority of Paxlovid treatments occurred within the 5-day window.

Because Paxlovid prescription timing was very right-skewed, the median and quartiles were all zero. Instead, we added the percentage of treated patients prescribed Paxlovid on days 0 and 1 to the results section.

3. Weights

a. Treatment assignment was defined over a 5-day window or grace period. In statistical analysis, please expand on how the weights were estimated to account for the time-varying nature of the treatment assignment. Please specify if a logistic regression model or another approach was used to estimate the probability of treatment. Please describe who this model was specified: was it a model for the daily probability of receiving Paxlovid or a model for the probability of receiving Paxlovid within 5 days of COVID index date?

We added the following to the statistical analysis section: "We used a single logistic regression model to estimate each patient's propensity to receive outpatient Paxlovid within 5 days of their COVID-19 index date". Because the treatment grace period and outcome observation period do not overlap, each patient's treatment status is fixed by the time their outcome is observed, so it is not necessary to model treatment as time-varying.

b. Please explain how the weights were stabilized. Usually stabilized weights imply two models, one for the denominator (to adjust for confounding) and one in the numerator (mostly to improve precision).

We added in the statistical analysis section that we calculated stabilized weights as $proportion\ treated\ /\ propensity\ score$ for the treatment group and $proportion\ treated\ /\ (1\ -\ propensity\ score)$ for the control group. We also added a reference for this method.

c. My understanding is that the cumulative incidence curve was estimated by giving each individual a time-fixed weight equal (or proportional) to the inverse of the probability of their observed Paxlovid treatment status during the 5-day window. If so, please expand in the statistical analysis section using this or similar language.

This understanding is correct. We expanded the sentence where we introduce the Aalen-Johansen estimators to clarify, and we think the changes made in response to your previous two comments also help to state this explicitly.

4. Confounders

a. Thank you for explaining the rationale for choosing the confounders! However, the variables mentioned in the text do not directly match the variables in the DAG. Consider updating the DAG.

We updated the DAG to use variable names that align better with the text. We also indicated which covariates were controlled for.

b. Please briefly report the distribution of the weights in results.

We added the median and standard deviation of weights to the results section.

5. Follow-up and censoring

a. When did follow-up start? At COVID-19 index date or 28 days after COVID-19 index date?

We added the following to clarify this. “We followed patients for 180 days following their COVID-19 index date. Follow-up started at day 0 and we started observing the outcome at day 29 to avoid attributing acute symptoms to PASC. Computable phenotype PASC predictions and GBD symptoms between days 0 and 28 were ignored. Censorship was possible from days 0 to 180.”

b. I don't think follow-up should be censored at a PASC occurring within 28 days given that the outcome is defined as PASC after 28 days. These events should just be discarded and Individuals who have PASC symptoms during the time window should be allowed to experience PASC after 28 days.

Thank you for pointing this out. We agree that this was a flaw in the study design. We now ignore PASC from days 0 to 28, and patients with those events remain at risk for PASC from day 29 on.

This change also had a side effect on our exclusion criterion for PASC prior to index. Before, we only considered each patient’s maximum computable phenotype prediction as their PASC onset date. If that score was above the threshold and before their index, we excluded them. But they could have had, for example, a CP prediction of 0.91 before index and a prediction of 0.99 after index, and stayed in the cohort.

Now, we separately consider scores from before index and from days 29-180. If patients have a CP score above the threshold prior to index, they are excluded, even if they have a higher score after index. This results in about 17k more patients excluded. Of course, these patients are very likely to have PASC, so the overall incidence rate estimates dropped substantially. However, this change did not affect our bottom-line conclusion.

c. Because there is no adjustment for censoring, all results rely on the strong assumption that censoring is non-informative, which means that individuals who were censored are a random sample of all included individuals. This is usually not the case. I suggest rerunning the analyses using censoring weights. Each individual would receive weights equal to the inverse of the probability of being uncensored.

We added IPCW to all analyses. We estimated IPC weights as you suggested and combined them with IPT weights to produce a single, time-fixed, combined weight for each individual, which was used in the Aalen-Johansen estimators.

d. Regarding death as a competing risk, thank you for clarifying that the focus is on the direct rather than the total effect. However, I recommend deleting the sentence 'The total effect would include any effect of Paxlovid treatment on PASC incidence that is mediated by death, which is less interpretable'. Technically this sentence is incorrect. Also, the direct effect is also problematic because 1) it quantified the effect of Paxlovid versus no Paxlovid in a world where patients cannot die and are immortal which is unrealistic and 2) it typically requires adjustment for selection bias. In the case of this article, I would say that choosing the direct or total effect does not matter much because mortality is relatively uncommon given the short follow-up.

   We revised the text related to our handling of death for clarity. Please see our response to the academic editor’s comment 3 for details.

e. Thank you for trying a doubly-robust approach in Sensitivity Analyses. I would change or delete the sentence 'Targeted maximum likelihood estimation was not feasible with our cohort and computing environment…'. TMLE is only one of many doubly robust methods. You could say 'Computer intensive doubly robust methods like target maximum likelihood estimation were not feasible with our computing environment. ' Notice that the hazard ratio estimated from the Cox model using IPW and baseline adjustment is not directly comparable with a risk ratio because hazard and risks are mathematically different.

   We have made these changes.

6. Target trial emulation (table 3)

a. Control treatment is not defined

   We added a control group definition and clarified the treatment group definition. Please see our response to the academic editor’s comment 2 and reviewer 2’s comment 2 for details.

b. Follow-up: text in the emulated trial is cut. Please specify the beginning and end of the follow-up. I don't see any mention here that follow-up was censored when PASC was recorded in the first 28 days. I see it is mentioned in Outcome, but it should be moved here. Here it is mentioned that individuals in the control group who received Paxlovid after day 5 are censored, but this was mentioned in the text!

   These details changed in the revision, as described above, but we made sure that all aspects of the protocol are mentioned in both the text and the table.

Minor Comments

1. I would avoid using the word 'significant' and 'unsignificant' throughout the text.

We removed all uses of this term.

2. Eligibility criteria (first paragraph): replace 'We excluded the period', with 'Individuals with COVID-19 diagnosis between December xxx '. Also, to improve clarify, I would move this sentence to the last paragraph of Eligibility criteria where all the exclusion criteria are listed.

   We revised this sentence to “We chose not to study the period between…” We prefer to keep it next to the sentence introducing the study period, because it is more related to that topic than to exclusion criteria.

3. The operational definition of the outcome, PASC, is reported twice in overview and treatment and outcome sections. Please consolidate.

   We condensed the method section substantially and moved most of the details to a supplement. We removed several repetitive chunks in the process. There was substantial repetition in the way the initial submission was structured, and we appreciate this suggestion, which greatly improved the flow of the paper.

4. Statistical analysis (second row): the author used the work 'rate'. Do they mean risk?

   Yes, thank you for catching this typo.

5. Also, in the DAG, all variables seem to be 'descendants' of age and demographics only. Therefore, adjusting for age and demographics would be sufficient to control confounding…

   We added the following to the figure title: “Note that this diagram only shows the relationships relevant to this study. Covariates may have other causes that are omitted for clarity.”

6. In statistical analysis, I recommend replacing 'We censored patients at the following events' with something like: 'Follow-up started at xx and ended at the earliest day of the outcome, death, last documented period, ect.'

   We updated this paragraph to include more details on the timeline. See our response to your comment 5a.

General Editorial Requests:

(Note: not all will apply to your paper, but please check each item carefully)

* We ask every co-author listed on the manuscript to fill in a contributing author statement, making sure to declare all competing interests. If any of the co-authors have not filled in the statement, we will remind them to do so when the paper is revised. If all statements are not completed in a timely fashion this could hold up the re-review process. If new competing interests are declared later in the revision process, this may also hold up the submission. Should there be a problem getting one of your co-authors to fill in a statement we will be in contact. Please do not add or remove authors without first discussing this with the handling editor. You can see our competing interests policy here: http://journals.plos.org/plosmedicine/s/competing-interests.

We will let all co-authors know to look out for this reminder.

* Please upload any figures associated with your paper as individual TIF or EPS files with 300dpi resolution at resubmission; please read our figure guidelines for more information on our requirements: http://journals.plos.org/plosmedicine/s/figures. While revising your submission, please upload your figure files to the PACE digital diagnostic tool, https://pacev2.apexcovantage.com/. PACE helps ensure that figures meet PLOS requirements. To use PACE, you must first register as a user. Then, login and navigate to the UPLOAD tab, where you will find detailed instructions on how to use the tool. If you encounter any issues or have any questions when using PACE, please email us at PLOSMedicine@plos.org.

We have uploaded TIF/EPS files for all figures other than Figure 2. We are still working to get the export to work for that figure. We uploaded a lower-quality version as a separate file in the meantime, and will provide the final figure during the editorial process if given the opportunity.

* Please ensure that the paper adheres to the PLOS Data Availability Policy (see http://journals.plos.org/plosmedicine/s/data-availability), which requires that all data underlying the study's findings be provided in a repository or as Supporting Information. For data residing with a third party, authors are required to provide instructions with contact information (web or email address) for obtaining the data. Please note that a study author cannot be the contact person for the data. PLOS journals do not allow statements supported by "data not shown" or "unpublished results." For such statements, authors must provide supporting data or cite public sources that include it.

We added a data availability statement that adheres to your policy (the same one used in our Paxlovid-hospitalization manuscript).

* We expect all researchers with submissions to PLOS in which author-generated code underpins the findings in the manuscript to make all author-generated code available without restrictions upon publication of the work. In cases where code is central to the manuscript, we may require the code to be made available as a condition of publication. Authors are responsible for ensuring that the code is reusable and well documented. Please make any custom code available, either as part of your data deposition or as a supplementary file. Please add a sentence to your data availability statement regarding any code used in the study, e.g. "The code used in the analysis is available from Github [URL] and archived in Zenodo [DOI link]" Please review our guidelines at https://journals.plos.org/plosmedicine/s/materials-software-and-code-sharing and ensure that your code is shared in a way that follows best practice and facilitates reproducibility and reuse. Because Github depositions can
be readily changed or deleted, we encourage you to make a permanent DOI'd copy (e.g. in Zenodo) and provide the URL.

We added a code availability statement that adheres to your policy (the same one used in our Paxlovid-hospitalization manuscript).

FORMATTING - GENERAL

* Abstract: Please structure your abstract using the PLOS Medicine headings (Background, Methods and Findings, Conclusions). Please combine the Methods and Findings sections into one section.

Our abstract is structured using these headings.

* At this stage, we ask that you include a short, non-technical Author Summary of your research to make findings accessible to a wide audience that includes both scientists and non-scientists. The Author Summary should immediately follow the Abstract in your revised manuscript. This text is subject to editorial change and should be distinct from the scientific abstract. Ideally each sub-heading should contain 2-3 single sentence, concise bullet points containing the most salient points from your study. In the final bullet point of 'What Do These Findings Mean?', please include the main limitations of the study in non-technical language. Please see our author guidelines for more information: https://journals.plos.org/plosmedicine/s/revising-your-manuscript#loc-author-summary.

We added an author summary.

* Please express the main results with 95% CIs as well as p values. When reporting p values please report as p<0.001 and where higher as the exact p value p=0.002, for example. Throughout, suggest reporting statistical information as follows to improve clarity for the reader "22% (95% CI [13%,28%]; p</=)". Please be sure to define all numerical values at first use.

We added p values and formatted CIs in this format.

*	Please include page numbers and line numbers in the manuscript file. Use continuous line numbers (do not restart the numbering on each page).

We added page and line numbering.

*	Please cite the reference numbers in square brackets. Citations should precede punctuation

We changed reference numbering to the square bracket format.

FIGURES AND TABLES

*	Please provide titles and legends for all figures and tables (including those in Supporting Information files).

All figures and tables have legends.

*	Please define all abbreviations used in each figure/table (including those in Supporting Information files).

All abbreviations used in figures and tables are defined in their titles.

*	Please consider avoiding the use of red and green in order to make your figure more accessible to those with color blindness.

We use colorblind friendly palettes in all figures.

SUPPLEMENTARY MATERIAL

In the published article, supporting information files are accessed only through a hyperlink attached to the captions. For this reason, you must list captions at the end of your manuscript file. You may include a caption within the supporting information file itself, as long as that caption is also provided in the manuscript file. Do not submit a separate caption file.

We added captions for the supplementary files at the end of the manuscript file.

When supplementary files are contained with a single file:
Please label the file as ‘S1 Supporting Information’.
Please apply alphabetical labelling to each table and figure contained within the S1 file. For example, ‘Fig A’ to ‘Fig Z’ and ‘Table A’ to ‘Table Z’.
Plain text does not need to be labelled and can just be given a title as necessary. For example, ‘Statistical Analysis Plan’.
Please cite tables/figures as ‘Fig A in S1 Supporting Information’ and/or ‘Table A in S1 Supporting Information’, for example.
Please cite plain text as, ‘Statistical Analysis Plan in S1 Supporting Information’, for example.

We labeled the supplementary file as such.

When supplementary files are uploaded as separate files:
Please label tables as ‘S1 Table’ (so on)
Please label figures as ‘S1 Fig’ (and so on)
Any additional documents (protocols/analysis plans etc.) can be labelled as ‘S1 Protocol’, for example.
Please cite items as exactly as labelled.”

REFERENCES

We formatted references per these guidelines.

* PLOS uses the numbered citation (citation-sequence) method and first six authors, et al.

* Please ensure that journal name abbreviations match those found in the National Center for Biotechnology Information (NCBI) databases (http://www.ncbi.nlm.nih.gov/nlmcatalog/journals), and are appropriately formatted and capitalised.

* Where website addresses are cited, please include the complete URL and specify the date of access (e.g. [accessed: 12/06/2023]).

* Please also see https://journals.plos.org/plosmedicine/s/submission-guidelines#loc-references for further details on reference formatting.

OBSERVATIONAL STUDIES

* Abstract: Please include the study design, population and setting, number of participants, years during which the study took place (enrollment and follow up), length of follow up, and main outcome measures.

We added length of follow-up, the only missing component, to the abstract.

* For all observational studies, in the manuscript text, please indicate: (1) the specific hypotheses you intended to test, (2) the analytical methods by which you planned to test them, (3) the analyses you actually performed, and (4) when reported analyses differ from those that were planned, transparent explanations for differences that affect the reliability of the study's results. If a reported analysis was performed based on an interesting but unanticipated pattern in the data, please be clear that the analysis was data driven.

We added a specific hypothesis and stated that the analytical methods did not differ from those planned.

*	Please state in the Methods section whether the study had a prospective protocol or analysis plan. If a prospective analysis plan (from your funding proposal, IRB or other ethics committee submission, study protocol, or other planning document written before analyzing the data) was used in designing the study, please include the relevant document(s) with your revised manuscript as a Supporting Information file to be published alongside your study and cite it in the Methods section. A legend for this file should be included at the end of your manuscript. If no such document exists, please make sure that the Methods section transparently describes when analyses were planned, and when/why any data-driven changes to analyses took place. Changes in the analysis, including those made in response to peer review comments, should be identified as such in the Methods section of the paper, with rationale.

The study did not have a prospective protocol, which we now state in the manuscript.
