# Supplementary material for: Effect of Paxlovid treatment during acute COVID-19 on Long COVID onset: An EHR-based target trial emulation from the N3C and RECOVER consortia
Source: PLoS Med. 2025 Sep 15;22(9):e1004711. doi: 10.1371/journal.pmed.1004711 (PMC12445499; doi:10.1371/journal.pmed.1004711)
Supplement: Attachment — Submitted filename: 02_Response to Reviewers Paxlovid PASC PLOSMed (Jan 2025).pdf [file pmed.1004711.s006.pdf]

Academic Editor:

The treatment strategies are still not well identified, and I think this has to do with the lack of a very discrete time zero and the use of a grace period. Currently, some post time-zero events are used to categorize individuals (e.g., if they are hospitalized in the first 5 days). Paxlovid in a non-hospitalized setting vs hospitalized setting is not a discrete distinction of treatment strategies (i.e., one would never randomize people to receive paxlovid in the hospital vs. not in the hospital…the selection bias in the different treatment arms would not make estimates very useful). Biologically, paxlovid works the same, but how sick the patient is, etc, may certainly moderate effects. In particular, "patients who received Paxlovid within 5 days, but during a hospitalization, were placed in the control group" is problematic. Hospitalization can be included in the overall eligibility criteria, but post time zero hospitalization can't be used to categorize exposure. It really does not make any sense to define exposure as paxlovid treatment within 5 days but then categorize people who were treated in the first 5 days but hospitalized as control. I recognize "in outpatient setting" is included in the definition, but this has no relevance clinically. No one would ever hospitalize or not hospitalize someone in order to give paxlovid to follow this definition. If distinguishing the effect modification by hospitalization is of interest, the analysis has to be designed differently. One way for the authors to address this is simply to have day 5 after treatment as time zero. Individuals who are not hospitalized are then categorized as to whether they have initiated paxlovid or not at that time point (individuals who are hospitalized within the first 5 days are excluded). However, this approach could create selection bias if there are differences in hospitalization that occur in those first 5 days. This would argue for the cloning-censoring approach, where treatment regimes can be very clearly specified and individuals can be under observation or censored based on what treatment regime is being assessed. The authors say there is no issue with immortal time bias, but the way they handled censoring certainly introduces it (i.e., to be exposed to intervention you need to stay out of hospital for at least 5 days and that is not the case in the control). I really do think the authors should consider this approach (even though findings may not change dramatically).

> Thank you for continuing to push this point. As you said in the last revision, it's important that the methods are airtight, even if they don't change the conclusion, especially for this topic. We are confident we have it right this time around.
>
> After considering all the options, we chose to address these interconnected issues by emulating a series of sequential trials. For each day *d* in the five-day treatment grace period, we emulate a trial where $t_0 = d$. Treatment is defined as a Paxlovid prescription on day *d.* We reassess the following exclusion criteria for each sequential trial:
>
> - Patient is alive at day *d*
> - Patient has not been hospitalized between covid index and day *d*
> - Patient has not received Paxlovid between covid index and day *d-1*

This approach resolves all of your concerns:

- No information after t0 is used to categorize patients into treatment groups.
- Hospitalization status is no longer an aspect of the treatment definition.
- There is no selection bias due to differences in hospitalization by treatment group, because patients who are hospitalized between t1 and t5 remain in the sequential trials for previous days.
- There is no immortal time bias, because treatment exposure for each sequential trial is based only on information from t0.

It is good to see that there is a supplemental analysis where deaths were treated as a competing risk and results were similar. I think this should be primary, which should not be difficult as the authors are already using the aalen-johansen approach. The question of interest is if 100 people are treated vs. 100 people are not, what happens to those 100 people. If there are differences in deaths, that is part of the story. Censoring deaths essentially changes the denominator in which you are assessing PASC. Treating it as a competing helps to maintain the denominator as 100. I would strongly argue this is the estimate of most interest, rather than trying to control for the aspect that is mediated by death.

We switched to treating death as a competing risk in the primary analyses, and we added a sensitivity analysis in which we treat death as a censoring event (formerly the primary analysis).

A lot of this discussion on the epidemiologic methods and rationale is in the supplement but is not even referred to in the main text. A reader would not even know that they should check the supplement for relevant details.

In the previous revision, one of the reviewers suggested we move more details to the supplement, so we are striking a delicate balance here. We took another pass at this and moved some of the details necessary to understand the study back to the body.

Although stratified estimates may take some time, they are still relevant and important to do (and extensions to complete and comprehensive revision can be granted).

We added stratified analyses for age and charlson comorbidity index.

Reviewer #1:

In the responses to my comments, the authors stated that the analyses have now been updated to include 500 bootstrap samples. However, the manuscript still says 200 bootstrap samples. Was this updated? And please make sure all the changes outlined in the responses are followed through in the manuscript.

For the previous revision, because the results were effectively identical with 200 and 500 bootstrap samples, we chose to use 200 samples throughout, rather than explain in the manuscript why we used 500 samples for the primary analysis and 200 for others.

However, this time around, we went ahead and ran all analyses with 500 samples so that we can state as much in the manuscript.

In the response to original comment 4, authors state "The immortal time period from days 0 to 28 is the same regardless of treatment assignment, so it does not lead to immortal time bias.". But this is not true. Even though there is the same period of time, there will be bias if the risk of the outcome or death in the 0-28 day period is different. At the very least, please can the authors describe the proportion of individuals with an outcome or who died between days 5 and 29 in each group. This will give us a better understanding of if there is bias.

This response addresses two components of your comment (outcome and death) separately.

First, in response to “there will be bias if the risk of **the outcome** in the 0-28 day period is different”. Our changes from the previous revision resolved this concern. The risk of outcome in the 0-28 day period is 0 for both groups. In the first submission, we censored for PASC in that period, which was problematic. We no longer do that, and we only measure PASC starting at day 29.

Second, in response to “there will be bias if the risk of **death** in the 0-28 day period is different”. We addressed this in the previous revision by adding IPCW. However, in this revision, we now treat death as a competing risk (see our response to your following comment). Now, any effects on PASC incidence from differential mortality risk are included in the estimate of Paxlovid’s total effect. This is equally true for deaths from days 0-28 and from day 29 onward.

We also added rates of death and censoring events from days 0-28 and days 29-180, by treatment group, to Table 1.

In the censoring models, what is defined as a censoring event? Death should not be included, as it is not "censoring". The final two options are last visit to the EHR and 180 days after index. This censoring only needs to be adjusted for if the authors believe the is a systematic difference between the groups in the risk of censoring due to these reasons. I struggle to see this. It would be interesting if the authors could also do a sensitivity analysis where they include results without the IPC weights to understand how sensitive results are to this analytical strategy.

This response also addresses two components of your comment (death and last visit) separately.

First, in response to **death** as a censoring event. In previous versions, we treated death as a censoring event in order to estimate the direct effect of Paxlovid treatment on PASC incidence, rather than the total effect (see Young et al 2020, https://doi.org/10.1002/sim.8471). We included a sensitivity analysis in which death was treated as a competing risk, and results were similar.

For this revision, we switched to treating death as a competing risk in the primary analyses, and we added a sensitivity analysis in which we treat death as a censoring event (formerly the primary analysis).

Second, in response to the **last visit** as a censoring event. Censoring due to loss to follow-up varies between treatment groups (7.9% among treated and 13.3% among untreated). We hypothesize that this is due to different baseline rates of healthcare utilization (treated patients have more visits, on average). We added IPCW in the previous revision to address this informative censoring. We also added a sensitivity analysis in which we do not censor at the last visit. Censoring at the last visit assumes that the last visit in each patient's EHR constitutes loss to follow-up, and that patients could have gotten care elsewhere. The added sensitivity analysis instead assumes that patients did not get care in the time after their last visit.
