# Supplementary material for: Effect of Paxlovid treatment during acute COVID-19 on Long COVID onset: An EHR-based target trial emulation from the N3C and RECOVER consortia
Source: PLoS Med. 2025 Sep 15;22(9):e1004711. doi: 10.1371/journal.pmed.1004711 (PMC12445499; doi:10.1371/journal.pmed.1004711)
Supplement: Attachment — Submitted filename: 02_Response to Reviewers Paxlovid PASC PLOSMed Rev3.pdf [file pmed.1004711.s007.pdf]

Academic Editor:

The authors have done a thorough job addressing the prior comments and it is much appreciated. One small query. Is it possible to include vaccination status as a stratified analysis? This would help make the manuscript more comprehensive as this has come up in prior literature as an effect modifier. The intent is not necessarily that the analysis is discussed in much detail, but just having the stratified analysis as an exploratory look at potential effect modifiers.

> We added vaccination status as a stratified analysis. We found no significant difference in Paxlovid's effect on PASC incidence between unvaccinated and fully vaccinated patients. This aligns with our prior work, published at PLOS Medicine, studying Paxlovid's effect on hospitalization and death. Although Paxlovid's relative effect varied by vaccination status, its absolute effect did not. We interpreted this as suggesting "that the benefit of Paxlovid in preventing hospitalization is similar for both vaccinated and unvaccinated populations [due to] the additive nature of the protective effects of vaccination and Paxlovid, rather than a multiplicative effect." Given our primary finding that Paxlovid has no significant effect on PASC incidence, these findings are consistent with that additive interpretation, as $a - b = (a + 0) - (b + 0)$.

I note that the authors briefly mentioned in the Methods section that ascertaining vaccination status in the EHR can be challenging. If they truly believe it cannot be done, it would be helpful for them to address this issue in the limitations section.

> We added the following to the limitations section: "COVID-19 vaccination is often undocumented in the EHR, because vaccination at pharmacies and other care sites is common. Our vaccination subanalysis addressed this issue by focusing on sites with reliable vaccination data."

Editors:

GENERAL

* Please confirm that your title complies with PLOS Medicine's style. Your title must be nondeclarative and not a question. It should begin with main concept if possible. "Effect of" should be used only if causality can be inferred, i.e., for an RCT. Please place the study design ("A randomized controlled trial," "A retrospective study," "A modelling study," etc.) in the subtitle (i.e., after a colon).

> We reviewed the manuscript and believe this requirement was met in the last revision. The study design is "an EHR-based target trial emulation". This design allows us to infer causality under a specific set of assumptions, which we describe in detail in the paper.

* Statistical reporting: Please revise throughout the manuscript, including tables and figures.
- Please report statistical information as follows to improve clarity for the reader ""22% (95% CI [13,28]; p</=)"".
- Please separate upper and lower bounds with commas instead of hyphens as the latter can be confused with reporting of negative values.
- Please repeat statistical definitions (HR, CI etc.) for each set of parentheses.

> We reviewed the manuscript and believe these requirements were met in the last revision.

* Please include the statement on code availability in the data availability statement.

> We moved discussion of code availability to the data availability statement and consolidated redundant aspects.

* Please ensure that all abbreviations are defined at first use throughout the text (including statistical abbreviations). Please also check figures and tables.

> We identified several places in the text and figures where abbreviation definitions were missing or out of order. We rectified these throughout.

* Please ensure that tables and figures, including those in supplementary files, are appropriately referenced in the main text.

> We reviewed the manuscript and believe this requirement was met in the last revision.

* Please review your text for claims of novelty or primacy (e.g. 'for the first time' or ‘novel’) and remove this language.

> We reviewed the manuscript and believe this requirement was met in the last revision.

* Please check that any use of statistical terms (such as trend or significant) are supported by the data, and if not please remove them. The term trend should be used only when the test for trend has been conducted.

> We reviewed the manuscript and believe this requirement was met in the last revision.

* Please include page numbers and line numbers in the manuscript file. Use continuous line numbers (do not restart the numbering on each page).

> We added continuous page numbers to the manuscript file.

ABSTRACT

* Please confirm that your abstract complies with our requirements, including providing all the information relevant to this study type
https://journals.plos.org/plosmedicine/s/submission-guidelines#loc-abstract

We reviewed the manuscript and believe this requirement was met in the last revision.

* Abstract Background: The final sentence should clearly state the study question.

We revised the final sentence to focus more clearly on this study's research question.

* Abstract Methods and Findings: We suggest clearly stating the primary and secondary outcomes.

We revised the abstract text to use the terms "primary outcome" and "secondary outcomes".

* When reporting age, please include a unit (i.e., years).

We added "years" to all mentions of age ranges throughout the manuscript.

* When describing the results, please mention that they are being compared to those of the control group.

We added this at the first mention of results in the abstract.

* Please include the study setting, years during which the study took place, and baseline demographics.

We reviewed the manuscript and believe this requirement was met in the last revision.

* Please include the important dependent variables that are adjusted for in the analyses (or describe the overarching categories).

We added overarching categories of covariates to the abstract.

* Please ensure that all numbers presented in the abstract are present and identical to numbers presented in the main manuscript text.

We reviewed the manuscript and believe this requirement was met in the last revision.

* In the last sentence of the Abstract Methods and Findings section, please describe the main limitation(s) of the study's methodology.

We added the following as the final sentence of this section: “This study’s main limitation is that the causal interpretation relies on the assumption that we controlled for all confounding variables.”

INTRODUCTION

* Please conclude the Introduction with a clear description of the study question or hypothesis.

We moved the first two sentences of Methods to the end of Introduction to provide a clear description of the research question and hypothesis.

METHODS AND RESULTS

* Please ensure to cite the STROBE checklist. When completing the checklist, please use section and paragraph numbers, rather than text excerpts.

* Figure 1: We suggest defining U07.1 diagnosis in the figure description.

Added.

* Figures and Tables: Please ensure that all abbreviations are defined in figure/table descriptions.

We reviewed all tables and figures and added missing abbreviation definitions for several of them.

* Are the results in Figure 3 repetitive of Table 3?

Yes, the relative risk ratios and p values in Figure 3 are also included in Table 3. Visualizing these results will help readers to see which results are significant and to compare confidence intervals more easily.

* “Among the age strata, Paxlovid had a significant effect on overall PASC onset only among patients aged 65+.” – what about the results for age group Age 18-24 and Age 35-49?

Thank you for catching this omission. We now mention this in the sensitivity analyses subsection of results and interpret it in the discussion section.

* What about the finding that there was an anti-protective effect in the sensitivity analysis for Positive Lab-only Index Events?

Thank you for catching this omission. We now mention this in the sensitivity analyses subsection of results and interpret it in the discussion section.

* Please include the Ethics Statement in the Methods section.

We moved the ethics statement to the end of the methods section.

* Table 1: Please state in the Table that the results are ‘N (%)’.

We added this to the table headers and description.

* Table 3: For the columns ‘Relative Risk’ and ‘ Absolute Risk Difference’, please include ‘(95% CI)’ in the column headers.

Added.
